# Supplementary material for: Evolution of brilliant iridescent feather nanostructures
Source: eLife. 2021 Dec 21;10:e71179. doi: 10.7554/eLife.71179 (PMC8691833; doi:10.7554/eLife.71179)
Supplement: Supplementary file 1. — (a) Result of phylogenetic pairwise t-test for difference in melanin layer thickness. P values corrected for multiple comparisons. (b) Phylogenetic signal for traits used in phylogenetic t-tests and ANOVA. (c) Summary statistics for brightness and saturation of optical model data, subdivided by melanosome type (used in linear models). (d) Summary statistics for brightness and saturation of plumage data, subdivided by melanosome type (used in Bayesian linear models). (e) Color diversity and saturation for optical model data using the UVS cone sensitivity function. (f) Color diversity and saturation for plumage data using the UVS cone sensitivity function. (g) Summary of results for linear model of saturation for optical model data. Model: Saturation (r.vec)~ hollow + thin+ platelet + hollow*platelet. Residual standard error: 0.072 on 4495 degrees of freedom. Multiple R-squared: 0.170, adjusted R-squared: 0.169. (h) Summary of results for linear model of brightness (double cone quantum catch) for optical model data. Model: Log brightness (double cone quantum catch)~ hollow + thin+ platelet + hollow*platelet. Residual standard error: 0.502 on 4495 degrees of freedom. Multiple R-squared: 0.459, adjusted R-squared: 0.459. (i) Summary of results for linear model of brightness (peak reflectance) for model data. Model: Log brightness (peak reflectance)~ hollow + thin+ platelet + hollow*platelet. Residual standard error: 0.4629 on 4495 degrees of freedom. Multiple R-squared: 0.540, adjusted R-squared: 0.540. (j) Summary of results for Bayesian linear model of saturation for plumage data. Model: saturation (r.vec)~ hollow + thin+ platelet + hollow*platelet+ PC. (k) Summary of results for Bayesian linear model of brightness (double cone quantum catch) for plumage data. Model: Log brightness (double cone quantum catch)~ hollow + thin+ platelet + hollow*platelet+ PC. (l) Summary of results for Bayesian linear model of brightness (peak reflectance) for plumage data. Model: Log br [file elife-71179-supp1.docx]

**Supplementary File 1**

**Supplementary File 1a**.

|  | thick solid rod | thin solid rod | hollow rod | solid platelet | hollow platelet | solid rod black feather |
| --- | --- | --- | --- | --- | --- | --- |
| thick solid rod | 1 | 0.0015 | 0.0015 | 0.0015 | 0.0015 | 0.0455 |
| thin solid rod | 0.0015 | 1 | 0.0015 | 0.2319 | 0.0048 | 0.0015 |
| hollow rod | 0.0015 | 0.0015 | 1 | 0.3862 | 0.5318 | 0.0015 |
| solid platelet | 0.0015 | 0.2319 | 0.3862 | 1 | 0.1608 | 0.0015 |
| hollow platelet | 0.0015 | 0.0048 | 0.5318 | 0.1608 | 1 | 0.0015 |
| solid rod black feather | 0.0455 | 0.0015 | 0.0015 | 0.0015 | 0.0015 | 1 |

**Supplementary File 1b**.

| Trait | Pagel's lamda | p |
| --- | --- | --- |
| Melanin layer | 0.96 | <0.001 |
| Air (diameter) | 0.56 | 0.001 |
| Number of layers | 0.81 | <0.001 |

**Supplementary File 1c**.

| Melanosome type | Mean saturation (vs cone) | Mean brightness | Mean peak reflectance (%) |
| --- | --- | --- | --- |
| Thick solid rod | 0.090 | 0.054 | 11.58 |
| Thin solid rod | 0.149 | 0.075 | 19.93 |
| Hollow rod | 0.143 | 0.097 | 26.71 |
| Solid platelet | 0.187 | 0.095 | 25.23 |
| Hollow platelet | 0.167 | 0.224 | 51.89 |

**Supplementary File 1d**.

| Melanosome type | Mean saturation (vs cone) | Mean brightness | Mean peak reflectance (%) |
| --- | --- | --- | --- |
| Thick solid rod | 0.093 | 0.072 | 9.68 |
| Thin solid rod | 0.142 | 0.119 | 20.63 |
| Hollow rod | 0.159 | 0.235 | 38.72 |
| Solid platelet | 0.144 | 0.237 | 43.23 |
| Hollow platelet | 0.194 | 0.215 | 35.20 |

**Supplementary File 1e**.

| Melanosome type | Mean color span | Occupied voxels | Mean saturation |
| --- | --- | --- | --- |
| Thick solid rod | 0.109 | 33 | 0.081 |
| Thin solid rod | 0.211 | 97 | 0.148 |
| Hollow rod | 0.207 | 100 | 0.145 |
| Solid platelet | 0.262 | 116 | 0.192 |
| Hollow platelet | 0.247 | 108 | 0.179 |

**Supplementary File 1f**.

| Melanosome type | Mean color span | Occupied voxels | Mean saturation |
| --- | --- | --- | --- |
| Thick solid rod | 0.154 | 13 | 0.132 |
| Thin solid rod | 0.225 | 22 | 0.163 |
| Hollow rod | 0.216 | 22 | 0.170 |
| Solid platelet | 0.204 | 25 | 0.151 |
| Hollow platelet | 0.237 | 24 | 0.201 |

**Supplementary File 1g**.

| Coefficient | Estimate | Std. Error | t value |
| --- | --- | --- | --- |
| thin | 0.059622 | 0.003392 | 17.575 |
| hollow | -0.006571 | 0.003392 | -1.937 |
| platelet | 0.037834 | 0.003392 | 11.152 |
| hollow*platelet | -0.014041 | 0.004798 | -2.927 |
| Intercept | 0.089683 | 0.002399 | 37.386 |

**Supplementary File 1h**.

| Coefficient | Estimate | Std. Error | t value |
| --- | --- | --- | --- |
| thin | 0.22949 | 0.02368 | 9.693 |
| hollow | 0.28932 | 0.02368 | 12.22 |
| platelet | 0.20454 | 0.02368 | 8.639 |
| hollow*platelet | 0.6386 | 0.03348 | 19.073 |
| Intercept | -2.97208 | 0.01674 | -177.528 |

**Supplementary File 1i**.

| Coefficient | Estimate | Std. Error | t value |
| --- | --- | --- | --- |
| thin | 0.46605 | 0.02182 | 21.36 |
| hollow | 0.34085 | 0.02182 | 15.62 |
| platelet | 0.22359 | 0.02182 | 10.25 |
| hollow*platelet | 0.5059 | 0.03086 | 16.39 |
| Intercept | 2.38295 | 0.01543 | 154.44 |

**Supplementary File 1j**.

|  | post.mean | lower 95% CI | upper 95% CI | pMCMC |
| --- | --- | --- | --- | --- |
| (Intercept) | 0.0802273 | 0.0132239 | 0.148827 | **0.023** |
| PC | 0.0853062 | 0.0456787 | 0.1259197 | **0.00012** |
| hollow | 0.0309548 | -0.0158424 | 0.078923 | 0.19776 |
| platelet | -0.0003642 | -0.0571464 | 0.0548228 | 0.98715 |
| thin | -0.0022319 | -0.0602206 | 0.0539252 | 0.93445 |
| hollow:platelet | 0.020505 | -0.0472965 | 0.0875612 | 0.5444 |

| Random effects | post. mean | lower 95% CI | upper 95% CI |
| --- | --- | --- | --- |
| phylogeny | 0.003508 | 0.001273 | 0.006187 |
| patch | 0.002388 | 0.001442 | 0.003419 |

|  | post. mean | lower 95% CI | upper 95% CI |
| --- | --- | --- | --- |
| Residual variance | 0.001387 | 0.00104 | 0.001775 |

**DIC: -722.8817**

**Supplementary File 1k**.

|  | post.mean | lower 95% CI | upper 95% CI | pMCMC |
| --- | --- | --- | --- | --- |
| (Intercept) | -2.76472 | -3.36719 | -2.15182 | **< 0.00001** |
| PC | 0.54649 | 0.17361 | 0.90788 | **0.00613** |
| hollow | 0.99645 | 0.54505 | 1.44248 | **< 0.00001** |
| platelet | 0.69742 | 0.18471 | 1.19531 | **0.00759** |
| thin | -0.30147 | -0.83223 | 0.24127 | 0.26653 |
| hollow:platelet | -0.52306 | -1.14786 | 0.08163 | 0.09451 |

| Random effects | post. mean | lower 95% CI | upper 95% CI |
| --- | --- | --- | --- |
| phylogeny | 0.2626 | 0.03862 | 0.5218 |
| patch | 0.2403 | 0.1441 | 0.3436 |

|  | post. mean | lower 95% CI | upper 95% CI |
| --- | --- | --- | --- |
| Residual variance | 0.07479 | 0.05483 | 0.09613 |

**DIC: 148.4126**

**Supplementary File 1l**.

|  | post.mean | lower 95% CI | upper 95% CI | pMCMC |
| --- | --- | --- | --- | --- |
| (Intercept) | 1.9479 | 1.3087 | 2.5866 | **<0.00001** |
| PC | 0.759 | 0.4074 | 1.1127 | **0.00018** |
| hollow | 1.1177 | 0.6987 | 1.5452 | **<0.00001** |
| platelet | 0.7802 | 0.2841 | 1.2764 | **0.00226** |
| thin | -0.1655 | -0.649 | 0.3212 | 0.50032 |
| hollow:platelet | -0.7032 | -1.3124 | -0.12 | 0.02178 |

| Random effects | post. mean | lower 95% CI | upper 95% CI |
| --- | --- | --- | --- |
| phylogeny | 0.3539 | 0.114 | 0.6246 |
| patch | 0.1433 | 0.07449 | 0.2185 |

|  | post. mean | lower 95% CI | upper 95% CI |
| --- | --- | --- | --- |
| Residual variance | 0.08233 | 0.06068 | 0.1064 |

**DIC: 163.4472**

**Supplementary File 1m.**

| Creator | Source link | License | License link |
| --- | --- | --- | --- |
| Yuri B | https://pixabay.com/de/vectors/feder-blau-fallen-federbusch-1689331/ | Pixabay licence | https://pixabay.com/service/license/ |

**Supplementary File 1n**.

| Species | Creator | Source link | License | License link |
| --- | --- | --- | --- | --- |
| Brown-headed cowbird (*Molothrus ater*) | ksblack99 | https://www.flickr.com/photos/ksblack99/27839803094/in/photolist-Jq7gx5-2gvEQaq-9gM5NB-6k3bok-sXMf1i-s1KsCE-wLLtpq-UJzqLj-tHYN2a-cZRhrQ-ottCRD-xtAvqR-otjy7M-tmX1Th-ot7h1D-wLM1UE-VjRCWR-sYrSTz-YhTFJt-fcw7GC-triMQq-otf89N-2j1LWqd-cyFiEU-xFSLGt-otmBtm-d9ZYVP-xbYXyf-wYFHYW-nkP1sk-niLgFH-ow8pH3-9RmJek-bWuhNF-xp5Hfu-e2q5JG-xyhRin-xmAt8R-otQYWd-oxhJSH-6ocTZH-xxsvY3-xpc7Ux-8Zb38Y-xDyNVf-xDL47Q-fcgRcB-fcw9B7-8p4HDu-8D2rJu | Public Domain Dedication 1.0 License | https://creativecommons.org/publicdomain/zero/1.0/ |
| Nicobar pigeon (*Caloenas nicobarica*) | Vassil | https://commons.wikimedia.org/wiki/File:Caloenas_nicobarica_Parc_des_Oiseaux_21_10_2015_1.jpg | Public Domain Dedication 1.0 License | https://creativecommons.org/publicdomain/zero/1.0/) |
| Elegant trogon (*Trogon elegans*) | Alan Schimierer | https://www.flickr.com/photos/sloalan/23784019851/in/photolist-CeHjtM-xDSVzM-ThPQxJ-f16DMA-sDvHz8-255o9zm-26JkLDQ-SAomTQ-Mprzxb-2jifF3j-FWBmSw-29sZoJ1-29fBGJC-FT3y6x-q2JuPh-BNurCF-27AjoxU-TyC1qT-SYEwZd-q4EdUT-eZRhrk-TyC1fH-f16EcY-pMsNA6-eip1qD-eip1Cx-bdUeZa-f16FBC-BUSvyY-eZRfHg-mfK6MK-wLKEf1-97vAfz-trttis-obWYBH-mcorU9-mcnpEa-mcnq3z-eZRm6X-s4xMei-s4qVr5-sMbdjf-97vBri-9LWYu2-eip15i-otoWXP-tJ4Z76-owbiig-abCcwb-8D2YtL | Public Domain Dedication 1.0 License | https://creativecommons.org/publicdomain/zero/1.0/) |
| Variable sunbird (*Cinnyris venustus*) | Leonard A. Floyd | https://www.flickr.com/photos/51217537@N00/32313930413/in/photolist-aNi8vT-aNi8pZ-o1ooZ3-cWAKFA-RetkHg-wcEb4j-wirGqF | Public Domain Dedication 1.0 License | https://creativecommons.org/publicdomain/zero/1.0/) |
| Ruby-throated hummingbird (*Archilochus colubris*) | ksblack99 | https://www.flickr.com/photos/ksblack99/36807506921/in/photolist-Y5y6si-2agP137-R14ZdB-UNFU2C-XfnW2e-28RSmaQ-Vfjy4w-LYnf3q-VkGQnN-BTPYcZ-288kcNj-29oxRTB-Cskfbt-XZHMcb-RCVcRJ-Y5y7T4-kgLEvv-27KtGMj-Y2LDM3-Pdb7Nq-V7aAHa-27Q62ue-255mZGU-K1yonZ-Y5y7kk-KG6hLu-RTyb2A-UmySgH-2cqKc1X-uRqpjR-Vcjojj-WQ7ceX-tUjqCr-ejwh1h-2agNTPA-255iPfY-X1AF8m-9gMU1B-X9uzmq-YedUJE-SRvpLL-V7aBii-BrvipA-A2Ah4T-29uMJrT-U1v3C7-Jb4i3T-XFTKvw-2e5tAph-fswF1r | Public Domain Dedication 1.0 License | https://creativecommons.org/publicdomain/zero/1.0/) |

**Supplementary File 1o.**

| Species | Creator | Source link | License | License link |
| --- | --- | --- | --- | --- |
| *Phasanius colchius* by | Mattia Menchetti | http://phylopic.org/image/8b720ff5-8e5a-4640-babc-cc30d6495a84/ | Public Domain Dedication 1.0 License | https://creativecommons.org/publicdomain/zero/1.0/ |
| *Anas fulvigula* | Sharon Wegner-Larsen | http://phylopic.org/image/cf522e02-35cc-44f5-841c-0e642987c2e4/ | Public Domain Dedication 1.0 License | https://creativecommons.org/publicdomain/zero/1.0/) |
| Trogon | Ferran Sayol | http://phylopic.org/image/5eca6929-759a-482c-b29c-b78340c35143/ | Public Domain Dedication 1.0 License | https://creativecommons.org/publicdomain/zero/1.0/) |
| Hummingbird | Ferran Sayol | http://phylopic.org/image/2bf1e800-5384-45cd-a533-ac940b8eadd6/ | Public Domain Dedication 1.0 License | https://creativecommons.org/publicdomain/zero/1.0/) |
| Starling | Ferran Sayol | http://phylopic.org/image/54ba1b9e-7bd1-4248-9aac-7218f1b1d6f4/ | Public Domain Dedication 1.0 License | https://creativecommons.org/publicdomain/zero/1.0/) |
